# Skill (or lack thereof) of data-model fusion techniques to provide an early warning signal for an approaching tipping point

**DOI:** 10.1371/journal.pone.0191768

**Published:** 2018-02-01

**Authors:** Riddhi Singh, Julianne D. Quinn, Patrick M. Reed, Klaus Keller

**Affiliations:** 1 Department of Civil Engineering, Indian Institute of Technology Bombay, Powai, Maharashtra, India; 2 School of Civil and Environmental Engineering, Cornell University, Ithaca, NY, United States of America; 3 Department of Geosciences, The Pennsylvania State University, University Park, PA, United States of America; 4 Earth and Environmental Systems Institute, The Pennsylvania State University, University Park, PA, United States of America; 5 Department of Engineering and Public Policy, Carnegie Mellon University, Pittsburgh, PA, United States of America; University of Hyogo, JAPAN

## Abstract

Many coupled human-natural systems have the potential to exhibit a highly nonlinear threshold response to external forcings resulting in fast transitions to undesirable states (such as eutrophication in a lake). Often, there are considerable uncertainties that make identifying the threshold challenging. Thus, rapid learning is critical for guiding management actions to avoid abrupt transitions. Here, we adopt the shallow lake problem as a test case to compare the performance of four common data assimilation schemes to predict an approaching transition. In order to demonstrate the complex interactions between management strategies and the ability of the data assimilation schemes to predict eutrophication, we also analyze our results across two different management strategies governing phosphorus emissions into the shallow lake. The compared data assimilation schemes are: ensemble Kalman filtering (EnKF), particle filtering (PF), pre-calibration (PC), and Markov Chain Monte Carlo (MCMC) estimation. While differing in their core assumptions, each data assimilation scheme is based on Bayes’ theorem and updates prior beliefs about a system based on new information. For large computational investments, EnKF, PF and MCMC show similar skill in capturing the observed phosphorus in the lake (measured as expected root mean squared prediction error). EnKF, followed by PF, displays the highest learning rates at low computational cost, thus providing a more reliable signal of an impending transition. MCMC approaches the true probability of eutrophication only after a strong signal of an impending transition emerges from the observations. Overall, we find that learning rates are greatest near regions of abrupt transitions, posing a challenge to early learning and preemptive management of systems with such abrupt transitions.

## Introduction

The Earth system can respond with abrupt and often persistent changes in response to slow and potentially small forcings (e.g., [1]). Abrupt changes can result from highly nonlinear processes that can trigger transitions between stable states. Examples for systems that can show such transitions include the Greenland ice sheet, the North Atlantic thermohaline circulation, phosphorus concentrations in a lake, and financial markets [2–8]. Some of the new states are undesirable in terms of their ecological or economic consequences. Information feedbacks play a key role for adapting management strategies to better detect and reflect negative threshold impacts [3, 4, 9]. As a result, an important body of research related to learning has emerged that seeks to better understand how to detect a catastrophic transition to an undesirable state with sufficient lead time so that adequate management steps can be taken to avoid it. The value(s) of the forcing(s) at which this transition occurs depends upon the underlying physical processes, and is commonly referred to as the ‘tipping point’ or the ‘critical threshold’.

The design of strategies to manage such tipping points can hinge critically on confident and timely predictions of an approaching threshold response [3, 10, 11]. Achieving this predictive capability can pose nontrivial challenges. A large body of research has addressed the problem of how to identify ‘early warning signs’ of impending threshold transitions using time series of the variables of interest. Signs such as increasing variance, increasing autocorrelation, etc., generally suggest an approaching transition [12–17]. However, efforts to compare and contrast these early warning signs have not yet identified a single metric that can successfully detect an upcoming transition, indicating the need for alternative methods to predict approaching transitions [18–20]. Another constraint of this approach is the requirement of long time series for a robust indication of an impending transition, particularly for higher order statistical moments [20, 21]. For many systems, such long-term data sets may not be available. If long-term data sets are not available for detecting an approaching tipping point, a conceptual model of the system can be developed based on physical understanding and the available data. The available observations can be used to identify a representative model structure and associated parameters. These calibrated models can potentially be used to predict the response of the system to a range of possible forcings. Models also enable identification of management strategies that avoid undesirable transitions in the natural system [10, 22].

While promising, model-based prediction of tipping points has been studied quite sparsely in the ecological literature [23–25]. Arguably one of the best studied case studies in this area is the classic ‘shallow lake problem’ that presents a management problem wherein the decision makers need to identify pollution strategies for a lake with dual states: oligotrophic (desirable), and eutrophic (undesirable). The problem was originally formulated by [22], and has since been used as a test-case for a wide range of decision—analytical approaches [9, 26–32].

An advancement towards utilising model based predictions for decision making was the analysis by [9]. The work by [9] showed that updating an adaptive strategy based on observations of phosphorus results in poorly controlled fluctuations between eutrophic and oligotrophic lake states in the face of model uncertainty. This analysis provides important insights using a simple approach to update decision makers’ beliefs regarding model uncertainty when simulating the lake’s dynamics. Specifically, the study adopts a likelihood function representing beliefs about two possible model structures, both incorrect representations of assumed reality, that are updated using a Bayesian model averaging approach. The beliefs are associated with a utility function that guides the choice of a management strategy. Although simplicity allows for fast implementation and insights, this simple design is perhaps a contributing factor behind this management strategy’s failure to prevent eutrophication as it does not allow the modeler to learn about the true model structure. Even if the true model structure is known, uncertainty regarding the model parameters characterizing the lake dynamics may inhibit identification of strategies that prevent eutrophication.

Subsequent analyses further explored the impact of uncertainty in the knowledge of the lake problem’s critical threshold on the risk attitude of a modeled rational decision maker [26, 27]. These studies show that both ecological and economic parameters can interact in determining the optimal management strategy [26]. These analyses also suggest that uncertainty regarding parameters that define the eutrophication threshold considerably impact the identified optimal policy. Precautionary behavior of the decision maker can vary nonmonotonically with uncertainty in perceiving and managing the risks associated with crossing the eutrophication threshold [27]. As uncertainty regarding the location of the threshold increases, decision makers may first exhibit precautionary behavior by reducing emissions. But as this uncertainty becomes larger, decision makers may revert to high emissions in absence of knowledge whether their reductions will have a significant impact on the probability of crossing the threshold. While these studies make the case that uncertainty with respect to an approaching transition has a sizeable impact on management strategy identification, an issue that warrants further investigation is how well and fast the uncertainty can be reduced given new observations. More precisely, how skilled are typical data assimilation schemes in this situation? There remains a gap in the ecological literature regarding the application of data-model fusion techniques to the problem of predicting an impending transition.

Of course, there is a very large body of literature that explores the performance of data assimilation methods when tracking systems with multiple equilibria [33–36]. Three types of uncertainties are commonly focused on in such analyses: uncertainty from noisy measurements, the stochastic nature of the system (process noise), and lack of knowledge about the system’s characterization (i.e., parametric uncertainty). Parametric uncertainty can be dealt with in a dual state parameter estimation scheme, where forecasts of system states and the underlying model parameters used to compute them are both assumed to be initially uncertain. Each new set of observations are then used to sharpen their estimates [37, 38]. The ability of data assimilation schemes to learn about systems with multiple equilibria in the presence of parametric uncertainty remains, to a large extent, an open challenge. Here, we focus on the lake problem as a highly nonlinear dual state parameter estimation problem with both parametric and observational uncertainties. The parametric uncertainties directly translate to uncertainty regarding parameterized model estimates of the critical threshold.

We compare and contrast four frequently employed data assimilation schemes in terms of their ability to predict an approaching eutrophication of the lake. We set up synthetic test cases using two different anthropogenic pollution strategies for an irreversible lake, one that triggers eutrophication and another that does not. We then analyze the performance of the methods in terms of their ability to determine the true parameters of the lake model, and to predict the approaching transition in the case of lake undergoing eutrophication. Our main goal in this study is to assess how well the different data assimilation methods can predict a critical threshold. Future research will examine how this information can be used to inform the design of adaptive strategies.

## Materials and methods

### The lake model as a dual state system

**Fig 1:**
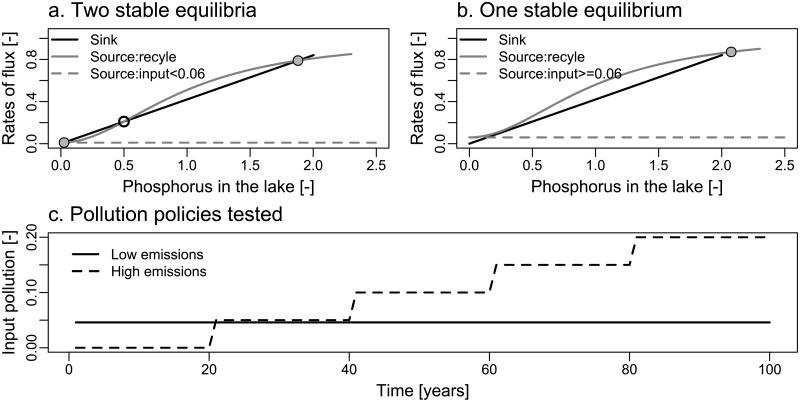
Dynamics of the irreversible lake. Dynamics of the irreversible lake that (a) displays two stable equilibria in absence of external inputs, and (b) only one stable equilibrium in presence of external inputs. Stable (unstable) equilibria are shown by filled (empty) circles. (c) Input pollution strategies with low (solid line) and high (dashed line) inputs of anthropogenic phosphorus in the lake.

We use the lake model to represent a system that can exhibit a sudden change in key states [22]. The lake model relates one state variable (*x*), two parameters (*b* and *q*), and, and an external input variable (*a*) as:

$$\begin{array}{c} X_{t+1}=X_{t}-bX_{t}+\frac{{X_{t}}^{q}}{1+{X_{t}}^{q}}+a_{t}, \end{array}$$ (1)

where *X*_*t*_ is the total phosphorus in the lake, and *a*_*t*_ is the anthropogenic phosphorus inputs to the lake at time *t*. The parameters of the lake model, *b* and *q* are referred to as the loss and recycle parameters, respectively. They determine the ability of the lake to remove and recycle phosphorus from the sediment, respectively. Certain combinations of these two parameters can result in an irreversible lake response. Irreversibility implies that the lake can potentially maintain an oligotrophic state at low pollutant concentrations and become irreversibly eutrophic after a threshold concentration of phosphorus is crossed. We call the phosphorus concentration beyond which the lake is irreversibly eutrophic the “critical phosphorus threshold”, or *P*_*crit*_. This is equal to the unstable equilibrium when *a*_*t*_ = 0 (Fig 1).

We illustrate the challenge of identifying the irreversible threshold using two anthropogenic pollution strategies, one that causes eutrophication while the other avoids it (Fig 1c). We term these strategies as the low emissions and high emissions strategies, respectively. Under the low emissions strategy, a constant amount of phosphorus is emitted at every time step, set at the highest possible value that still keeps the lake in an oligotrophic state. The high emissions strategy starts with no anthropogenic phosphorus emissions to the lake, but increases emissions every 20 years. These increases are such that the lake undergoes eutrophication toward the middle of the 100-year simulation period.

### Description of the data assimilation methods

Coupled human-natural systems are approximated via conceptualizations mathematically represented by equations. These equations comprise of constants (termed parameters) that represent time invariant characteristics of the system and state variables that evolve with time. The equations, together with the parameters, constitute the model structure that abstracts the system of interest. Typically, modelers are unaware of the true values of parameters that characterize the system but are able to formulate the model structure, which encapsulates the modeler’s understanding of the dominant physical processes in the system. Even though the exact values of parameters may not be known, modelers are usually able to define their feasible or physically meaningful ranges. Within this range, the true values of each parameter need to be identified so that reliable predictions of system states can be made. Observations of system states are used to guide parameter identification. Parameters that produce simulations closer to observations are more likely to represent the true system.

Bayes’ theorem provides a way to combine the modeler’s prior information about the state variables and parameters, with available observations of system states according to:

$$\begin{array}{c} p\left(x_{t},\Theta |y_{t}\right)∼p\left(y_{t}|x_{t},\Theta \right)p\left(x_{t},\Theta \right), \end{array}$$ (2)

where *x*_*t*_ and *y*_*t*_ are vectors of state variables and observations that vary with time, *t*, respectively; Θ is the parameter vector, *p*(*x*_*t*_, Θ|*y*_*t*_) is the posterior probability of parameters and state variables given the observations, *p*(*y*_*t*_|*x*_*t*_, Θ) is the likelihood, and *p*(*x*_*t*_, Θ) is the prior probability of parameters and state variables. Likelihood is the probability of observing phosphorus concentrations, *y*_*t*_, for a given combination of state variables (*x*_*t*_) and parameters (Θ). Parameters with higher likelihood values produce simulations closer to the observations and vice-versa. The prior probability distributions denote the belief of a modeler in the parameter value before a new observation becomes available. Bayes’ theorem combines the prior belief with observed data to update the probability of a parameter value being representative of the system. This updated probability distribution is termed the posterior.

In simple terms, data assimilation is the application of Bayes’ theorem to update information about the system states and parameters. In absence of any observations, the modeler will define prior probabilities for initial states of the system and parameters based on expert knowledge. Once the first set of observations are obtained, this prior will be updated to provide the posterior distribution of parameters and states. As more observations are obtained, the posterior at the previous time step acts as the prior for the current step. Here, we cast the data assimilation problem in a joint state-parameter estimation framework by appending unknown parameters at the end of the state variable vector. In order to do this, constant parameters are assumed to be time-varying by adding noise to them. This is a common way to estimate unknown parameters in filtering algorithms as additional observations become available [38, 39]. From here on, we represent this joint state-parameter vector as *J*: (*x*, Θ). There are two main steps that are repeated in each cycle of the data assimilation:

1. Time update of the model states: running the model using the updated state values from the previous time step:
  $$\begin{array}{c} p\left(J_{t}|y_{t}\right)=\int \;p\left(J_{t}|J_{t-1}\right)p\left(J_{t-1}|y_{t-1}\right)\;dJ\; \end{array}$$ (3)
  where *p*(*J*_*t* − 1_|*y*_*t* − 1_) is the probability of *J*_*t* − 1_ conditional on the measurements up to the last time step *y*_*t* − 1_, and *p*(*J*_*t*_|*J*_*t* − 1_) is the probability of *J*_*t*_ based on the updated value of *J*_*t* − 1_ at the last time step. In this step, the information about parameter and states conditional on observatoins up till the last time step, *t* − 1, is used to predict future states of the system.
2. Measurement update: Once new measurements, *y*_*t*_, are available at time step, *t*, they are used to estimate the posterior at time step, *t*, using:
  $$\begin{array}{c} p\left(J_{t}|y_{t}\right)=\frac{p\left(y_{t}|J_{t}\right)p\left(J_{t}|y_{t-1}\right)}{p(y_{t}|y_{t-1})}, \end{array}$$ (4)
  where *p*(*J*_*t*_|*y*_*t*_) is the probability of *J*_*t*_ conditional on the current measurement *y*_*t*_, *p*(*y*_*t*_|*J*_*t*_) is the probability of observing the measurement conditional on the state *J*_*t*_ that can be calculated by Eq (3), and *p*(*y*_*t*_|*y*_*t* − 1_) is the normalizing constant, calculated as:
  $$\begin{array}{c} p\left(y_{t}|y_{t-1}\right)=\int \;p\left(y_{t}|J_{t}\right)p\left(J_{t}|y_{t-1}\right)\;dJ. \end{array}$$ (5)

In order to exactly apply Eqs (3–5), the integral in Eq (5) should be analytically derived. This is not feasible in most real world examples and thus simplifications of Eqs (3–5) are applied via different types of recursive filters. Each filter makes assumptions about the type of model, errors in the model and measurement functions. To illustrate this, we present a generalized form of Eq 1:

$$\begin{array}{c} \left(x_{t},\Theta_{t}\right)=f\left(x_{t-1},\Theta_{t-1}\right)+B\left(a_{t}\right)+w, \end{array}$$ (6)

$$\begin{array}{c} y_{t}=h\left(x_{t}\right)+\nu , \end{array}$$ (7)

where *f*(⋅) represents the model function for simulating state variables subject to model parameters updated through time, *h*(⋅) represents the measurement function that applies on model state to convert them to measurable variables, *a*_*t*_ represents external inputs that act on the system through the matrix *B*, *w* represents the model (process) noise, and *ν* represents the measurement noise, and the remaining terms are defined before.

We compare and contrast three forms of recursive filters that implement Eqs (3–5)—the ensemble Kalman filter (EnKF), the particle filter (PF), and the Metropolis-Hastings algorithm which implements the Markov Chain Monte Carlo method (MCMC). In addition, we also test a rather simple screening method, Pre-calibration (PC) that searches the space of states and parameters to identify those combinations that satisfy a performance criterion [40]. Each method is discussed briefly below with details in S1 Text.

1. Pre-calibration (PC): Pre-calibration (PC) is a simple technique that constrains the space of state variables and parameters based on their predicted response using the rule:
  $$\begin{array}{c} M=\frac{1}{T}\sum_{t=1}^{T}\frac{|y_{t}-h\left(J_{t}\right)|}{2\nu }<1, \end{array}$$ (8)
  where *M* determines whether a given initial state and parameter vector is acceptable, and the remaining terms are defined before [40, 41]. For any combination of initial state and parameters, system trajectories are generated and *M* is estimated. If *M* is less than 1, the initial state and parameter values are accepted; otherwise, they are rejected. One can interpret this method in Bayesian terms with a likelihood function defined as:
  $$\begin{array}{c} p\left(y_{t}|J_{t}\right)=\{\begin{array}{c} 0,M>1\\ 1,M<=1. \end{array} \end{array}$$ (9)
2. The Ensemble Kalman filter (EnKF): EnKF approximates the posterior distribution in (2) through a randomly sampled set of realizations [42]. Random samples (ensemble members) are generated by assuming a prior distributions of state variables and parameters, which are updated sequentially using the observations. Using ensemble members enables EnKF to approximate highly nonlinear models, however, the errors should still follow a Gaussian distribution.
3. The particle filter (PF): This is the most general form of the Bayesian update recursion. The model can be linear or nonlinear and the errors can be Gaussian or otherwise. However, practical implementation of this filter often requires a simplification of Eqs 4 and 5. The continuous probability distributions are discretized by applying weights on randomly sampled particles to approximate true probabilities [39]. In addition, PF requires specification of a likelihood function to update weights based on the values of the measured variable.
4. Markov Chain Monte Carlo Method (MCMC): MCMC is implemented using the Metropolis-Hastings algorithm that samples the joint state-parameter space [43]. It can directly use the time and measurement(Eqs 3 and 4) without the need for calculating the integral in Eq 5. In order to facilitate a fair comparison with the recursive filters, we apply MCMC in a sequential manner, i.e., the posterior at each time step is estimated by providing MCMC observations until that time step.

### Application of the data assimilation methods

To simplify the comparison with previous studies, we follow a typical design [4]. First, true values of the initial phosphorus level in the lake and of the loss and recycle parameters are assumed. Only the phosphorus concentration in the lake is directly observable, simplifying the measurement function *h*(⋅). In the joint state space formulation, loss and recycle parameters constitute the parameter vector, Θ, that is appended to the state variable, *x*. The parameter settings are adapted from prior literature such that the lake shows an irreversible response to a strong forcing [22, 30–32]. Using the assumed true values of the model state and parameters, along with the input pollution strategies, we generate the assumed truth from which observations of phosphorus are generated by adding measurement noise (Figs A-B in S1 File). Hereafter, we refer to the simulations of the lake’s phosphorus concentrations generated from assumed true values of lake model parameters and initial phosphorus level in the lake as the ‘assumed truth’.

We apply data assimilation methods to the hypothetical observations to assess whether or not they are able to recover the true values of the model state and parameters. Each method starts with the same biased and uncertain guess of the initial conditions and parameters. We choose the guesses so that each method starts with mean values of parameters and initial phosphorus levels that imply a greater capacity to deal with anthropogenic pollution than the true capacity. Biases also arise due to the use of a log normal distribution in representing uncertainty in the parameters. The model’s stochastic noise, *w*, is set to zero, reducing the sources of uncertainty to initial condition uncertainty and measurement uncertainty. This assumption simplifies the implementation of the assimilation techniques and is often used in similar analyses [4, 44, 45]. This simplification also makes our framework a lower complexity case and if we find that learning here is challenging, additional uncertainties are likely to confound the performance of data assimilation methods further. The specifications of the biases and uncertainties in the model parameters and initial state, termed hyper-parameters for the problem, are listed in Tables 1 & 2.

**Table 1:** Parameters and initial conditions for the lake model.

Parameters	Assumed truth	Initial mean guess	Initial covariance guess
*b*	0.42	0.62	0.20
*q*	2.00	4.00	1.00
*X*_0_	0.05	0.20	0.20

Set up of the lake problem for generating the assumed truth. All values are dimensionless. Note that the setup is the same for both cases, as they differ only in the input pollution vector.

**Table 2:** Set up of the data assimilation problem.

Hyper-parameter	Value for both cases
Measurement variance [*ν*]	[0.01]
Process noise [*X*_0_, b, q]	[0 0 0]
Simulation time [T]	100 years
Time step	1 year

Hyper-parameters for the data assimilation problem. All values are dimensionless.

### Skill metrics for prediction of threshold based response

We assess the data assimilation methods in terms of their ability to:

1. simulate the time evolution of phosphorus close to the assumed truth,
2. identify the true values of model parameters, and,
3. predict an approaching transition. The performance of each simulation of phosphorus in the lake is quantified by calculating the root mean squared error (RMSE) between the estimated amount of phosphorus in the lake and the assumed truth:
  $$\begin{array}{c} RMSE=\sqrt{\frac{\sum_{t=1}^{T}{\left(x_{t}^{i}-x_{t}^{*}\right)}^{2}}{T}}, \end{array}$$ (10)

where *RMSE* is the root mean squared error across the time period of simulation, *T*, $x_{t}^{i}$ is the estimate of phosphorus in the lake by the *i*^*th*^ data assimilation scheme at time *t*, and, $x_{t}^{*}$ is the assumed truth. Since the performance of all data assimilation schemes is likely to depend upon the number of samples used for approximating the prior probabilities, we estimate RMSE values as a function of different numbers of function evaluations (NFEs) of the lake problem.

Following [4], we also identify the skill of data assimilation methods in identifying the true values of model parameters through the estimated probability distribution of parameters at the “point of no return”. The point of no return is the first year for which even complete reduction of anthropogenic emissions of phosphorus in the lake cannot avert eutrophication. The third assessment criterion quantifies the ability of each method to successfully predict the approaching eutrophication before the “point of no return”. Using ensembles updated by learning till the point of no return, we project the phosphorus levels in the lake for the future time steps, up to the 100^*th*^ year. The ratio of number of ensembles that predict eutrophication in the lake at year 100 to the total number of ensembles is termed as the probability of eutrophication.

Before presenting the results, we note a few additional methodological details. First, from here on, we use the general term ensemble members to refer to ensemble members of the EnKF, particles of the PF, and samples of MCMC and PC. Second, while applying EnKF, PF, and PC, we exclude those ensemble members that do not simulate physically realistic lake parameters or that simulate a negative phosphorus level. Note that the physically plausible ranges of lake parameters are set between 0–1, and 2–8, for the loss and recycle parameters, respectively [22]. This leads to deterioration in the number of ensemble members as learning time increases, the consequences of which are discussed in S2 Text. Next, the performance of each method is likely to vary with the random noise applied to assumed true phosphorus time series to generate synthetic observations. We thus compute performance across 10 random realizations of observed phosphorus (Figs A-B in S1 File). Finally, we use NFEs to quantify computational effort with each ensemble member being equivalent to 100 NFEs as the lake model runs for 100 time steps. Thus, a 1000-member ensemble of EnKF performs 1000 x 100 NFEs.

## Results

We first visually compare each method in terms of their simulated trajectories of phosphorus in the lake and estimates of model parameters. We then compare the methods in terms of the *R.M.S.E* between their simulated trajectories and the assumed truth. As all methods depend on the number of ensemble members chosen to approximate distributions, we further present the results across a range of ensemble sizes. We then explore the ability of each method to identify the true parameters of the lake model and their skill in predicting an impending eutrophication tipping point before the point of no return.

### Assessment of learning trajectories

**Fig 2:**
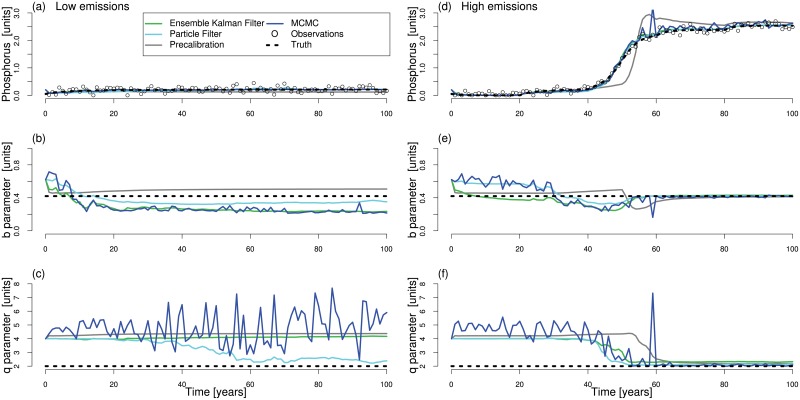
Mean trajectories from each data assimilation scheme. Mean learning trajectories (y-axis) for (a, d) phosphorus in the lake, (b, e) loss parameter, b, and (c, f) recycle parameter, q, as a function of learning time (x-axis) for (a-c) low emissions and (d-f) high emissions strategies. EnKF, PF, PC, and MCMC learning trajectories are shown by green, sky blue, gray, and dark blue lines, respectively. The assumed true values of parameters and resultant phosphorus trajectory are shown by dashed black lines. Empty black circles show the synthetic observations of phosphorus in the lake. Each data assimilation method uses 5 million NFEs, excluding overheads which differ for each method.

Visual inspection of the forecasted trajectories of the lake model’s state variables and parameters reveals a perhaps unexpected poor skill across all algorithms on the low emissions case, and mixed skill on the high emissions case (Fig 2). All methods are represented by their mean trajectories in Fig 2. EnKF, PF, PC, and MCMC methods simulate an ensemble of trajectories, but only their mean values are shown. We note that there are considerable uncertainties in the forecasts, and therefore present the 90% confidence intervals for each method in Figs C-N in S1 File. along with supporting text on estimation of these intervals in S3 Text.

None of the considered methods identify the model parameters accurately for the low emissions case, as shown by the large biases and fluctuations in estimated parameter values in Fig 2b–2c. For this case, PF gets closest to the true values of both parameters. In contrast, all methods identify both loss and recycle parameters with higher accuracy for the high emissions case (Fig 2e–2f). Additionally, EnKF and PF converge to true values of the loss and recycle parameters much faster than PC. MCMC also converges at a similar rate as EnKF and PF but displays an erratic behavior in estimates before convergence. This is likely due to the constraint on the MCMC sample size to ensure that each assimilation method was restricted to 50,000 NFEs. We find that increasing the sample size does reduce fluctuations in MCMC parameter estimates, but does not change their overall trends (Fig O in S1 File). Thus, any biases in parameter estimates remain similar.

**Fig 3:**
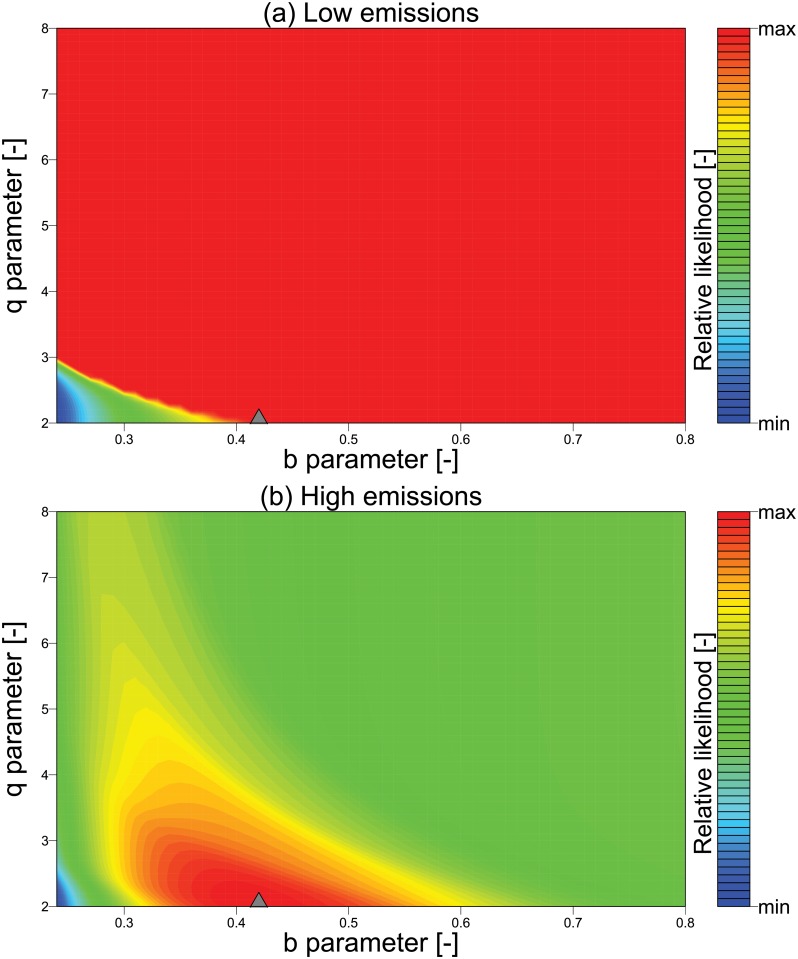
Likelihood surface for low and high emissions strategies. Colored contours of the conditional probability (likelihood) of the observed values of phosphorus in the lake given various combinations of loss, b, (x-axis) and recycle, q, (y-axis) parameters. The log likelihoods are estimated using all 100 observations for the (a) low, and (b) high emissions strategies. The star shows the location of assumed true values for the parameters.

Of note is the sudden convergence of several methods to the true parameter estimates toward the middle of the simulation period for the high emissions case (year 45 in Fig 2e–2f). We attribute this to an increase in the information content of observed phosphorus values as the lake approaches eutrophication which is evident in the Kalman gain estimates (Fig P in S1 File). In contrast, in a case without eutrophication the observations have very little information to guide the methods towards the correct parameter estimates. This can be visualized by examining the variation in the conditional probability, or the likelihood, of the observations across a range of parameter values. If a given parameter vector is unique such that it is the only vector that can result in the observed phosphorus values, the likelihood surface will show a sharp peak around the (assumed) true values. On the other hand, if the observations can result from a range of possible combinations of parameters, the likelihood surface will be relatively flat across that range of parameters. We find that the likelihood surface (Fig 3) has a distinct peak for the high emissions case but not for the low emissions case, explaining the poor performance across methods in the low emissions case.

### Performance metric based assessment

In applications of ensemble-based learning methods, the analyst is unlikely to know *a priori* the appropriate number of ensemble members to represent the probability distribution. Also, many applications employ models that are computationally expensive thus posing limitations on the number of ensemble members that can be used. Hence, we analyze the question: how does the performance of the considered ensemble based methods depend on the ensemble size?

**Fig 4:**
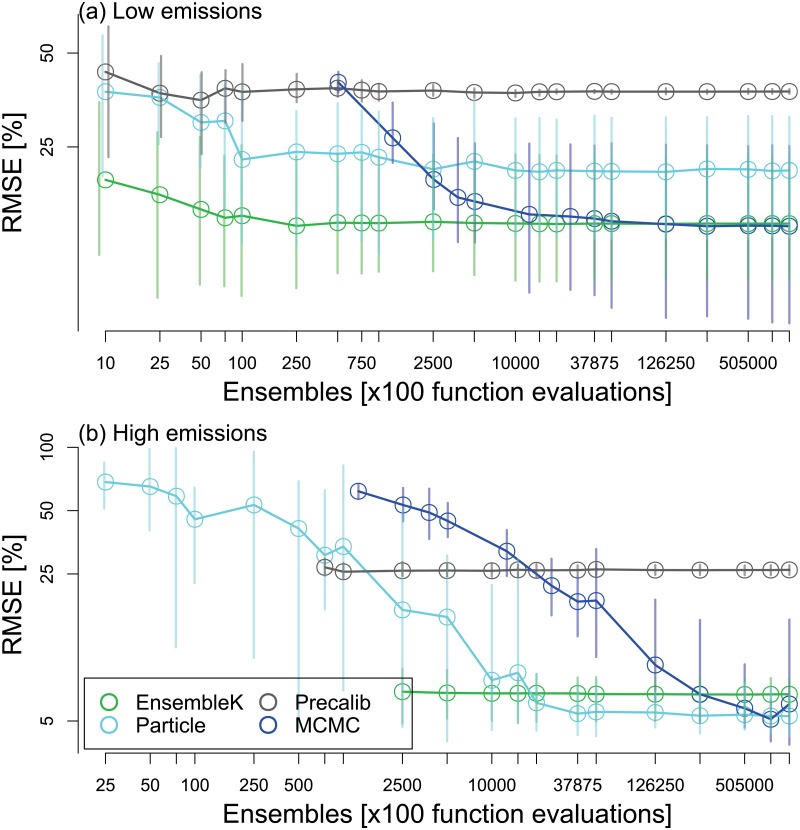
Performance as a function of ensemble size. Performance of four data assimilation methods as a function of the number of ensembles. The performance is expressed as root mean squared error (RMSE) between observed and forecasted phosphorus states scaled relative to the observed mean phosphorus levels across the entire time period (y-axis). RMSE is shown as a function of the number of ensembles, particles, samples, or chain length, together termed ensembles (x-axis), for the EnKF, PF, PC, and MCMC methods, respectively. Markers (vertical lines) show the mean (range) of RMSE estimates across 10 random sets of 100 year observations generated using the same assumed truth. Results are shown for (a) low, and (b) high emission strategies.

We find considerable variability in algorithmic performance as a function of ensemble size, indicating that the choice of data assimilation scheme and ensemble size does impact learning (Fig 4). PC performs poorly irrespective of the number of ensemble members for both cases and is not discussed further. We quantify performance by estimating the relative RMSE between the observed and forecasted phosphorus in the lake as estimated by each method against an increasing number of ensemble members (and NFEs). For smaller numbers of ensemble members, EnKF converges fastest to high performance for the low emissions case (Fig 4a). EnKF attains relative RMSE between 9%–23% across 10 random realizations with only 75 ensemble members. On the other hand, PF requires 250 ensemble members to converge to its best performance of 12%–33%. Further increasing the number of ensemble members from 500 to 20,000 has little effect on performance, with relative RMSE varying slightly between 11%–14% (best), and 31%–35% (worst) across random realizations. MCMC starts with poor RMSE performance ranging between 37%–44% for 505 ensemble members. However, as the ensemble size is increased to 12,625, MCMC converges to high performance regions, equivalent to EnKF. Overall, despite its approximate nature, EnKF maintains overall high performance even when compared with MCMC.

Although the overall dynamics of performance in the case of the high emissions strategy is similar to that observed above for the low emissions strategy, there are also some noticeable differences (Fig 4b). As observed for the low emissions case, PC emerges as the poorest performer overall, but in this case, PF and MCMC display poorer performance than PC for smaller ensemble sizes. Again, as with the low emissions case, EnKF displays fastest convergence to high performance, though at a much higher ensemble count of 2500 (as opposed to 75 in the prior case). An interesting feature of EnKF’s performance in the high emissions case is the lack of variation in its performance with ensemble size. EnKF’s performance ranges between 5%–9% for 2500 ensemble members and 5%–8% for a million ensemble members. As opposed to EnKF, PF and MCMC require relatively large ensemble sizes to converge to high performance. PF and MCMC outperform EnKF after 20,000 and 505,000 ensemble members, respectively. However, the performance differences are relatively low with relative RMSE between 5%–8%, 4.2%–8.4%, and 5%–29% for EnKF (ensemble sizes>20,000), PF (ensemble sizes>20,000), and MCMC (ensemble size>505,000), respectively. If computationally constrained, EnKF is most efficient. But its performance varies considerably for both low and high emission cases, thus necessitating a convergence analysis.

### Performance at the point of no return

**Fig 5:**
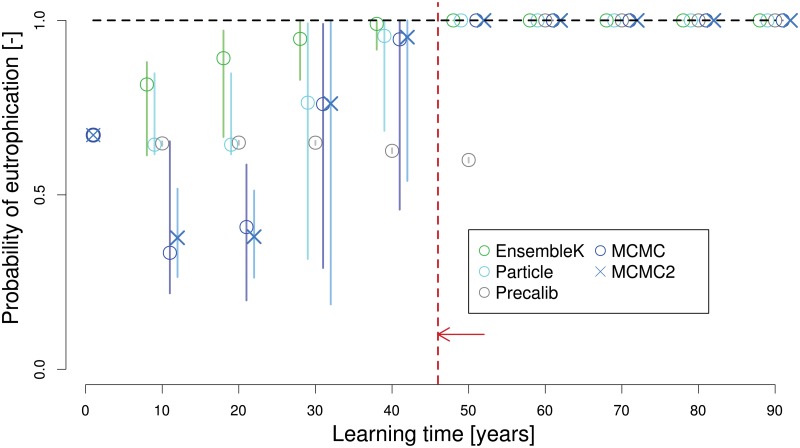
Probability of eutrophication as a function of learning time. Estimates of the probability of eutrophication of the lake (y-axis) as a function of learning time (x-axis) for each assimilation method. Markers (vertical lines) show the mean (range) of the probability estimates across 10 random sets of 100 year observations generated using the same assumed truth. The point of no return is marked at the 45th year, denoting the time after which even complete reduction in anthropogenic emissions of phosphorus will not avert eutrophication. Results are shown for the high emission strategy, hence the truth is set at a probability of eutrophication value of 1.

**Fig 6:**
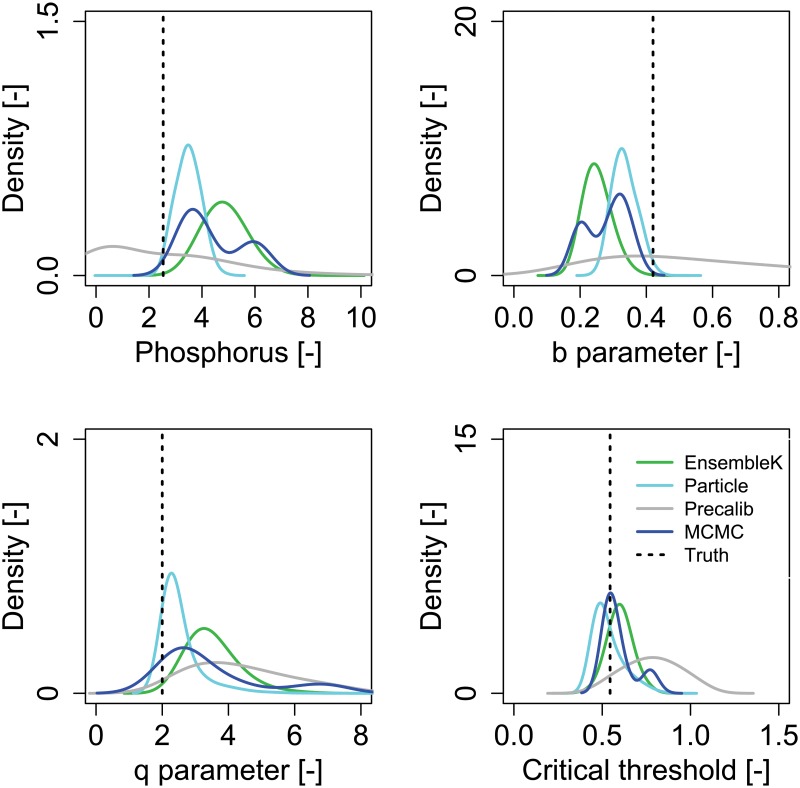
Estimates of model parameters at the point of no return. Probability density function of (a) phosphorus in the lake at the 100th year, (b) loss parameter, b, (c) recycle parameter, q, and, (d) critical threshold (*P*_*crit*_) as estimated by each assimilation method. The methods assimilate phosphorus observations until the 45th year, the year until which a complete reduction in anthropogenic phosphorus inputs will prevent eutrophication. Vertical solid lines represent the assumed true values of model parameters and resultant true values of the phosphorus in the lake at the 100th year. For each method, we use kernel density estimation to generate the PDFs from the ensemble members’ estimates. Results are shown for the high emission strategy.

The main goal of our analysis is to understand the ability (or lack thereof) of different data assimilation methods to reliably predict, with sufficient lead time, an impending transition to a eutrophic state. Thus, we now turn to a closer examination of the high emissions case in which an abrupt transition to an undesirable state occurs. We first examine the ability of each method to predict eutrophication of the lake as a function of different learning times (Fig 5). This is followed by an assessment of the ability of each method to converge to true estimates of model parameters before the point of no return (Fig 6). We estimate the point of no return as the 45th year, which is the time at which phosphorus concentrations in the lake exceed the critical phosphorus threshold (*P*_*crit*_ of 0.54), a value predetermined by the lake parameters.

Among all methods, EnKF emerges at the fastest learner in terms of its ability to predict a high probability of an impending eutrophication in the lake, thus enabling expedient management actions (Fig 5). We quantify this by estimating the probability of eutrophication as a function of learning time using each method. We simulate each ensemble member forward in time and estimate the probability of impending eutrophication as the fraction of ensemble members that predict eutrophication. We diagnose a eutrophic state once the phosphorus concentration exceeds *P*_*crit*_. As all methods start with the same distribution of model parameters, they have the same prior value of probability of eutrophication before any learning begins. This value is slightly higher than 0.50, as we assume lognormal distributions.

As each method assimilates the observations, EnKF and PF predict increasing probability of eutrophication, but for the first two decades MCMC’s estimates of the probability decrease. Between the second and third decades, MCMC’s estimates of the probability increase sharply and come close to those estimated by EnKF and PF (please refer to Fig Q in S1 File for a further analysis of MCMC’s convergence). This is also the time period in which the process of eutrophication begins as seen in (Fig 2d). The transition to a eutrophic state is almost complete around the middle of the time period (year 50). Overall, MCMC first gives a false signal of low chances of eutrophication for the chosen anthropogenic phosphorus pollution strategy, but corrects its estimates fairly quickly at the onset of eutrophication. This observation is consistent across increasing ensemble sizes for MCMC; the increase in the number of ensemble members reduces uncertainty but not the bias in the estimated probability of impending eutrophication. Between EnKF and PF, EnKF converges faster to the true probability of impending eutrophication (=1); however, due to variations across random seeds, none of the methods attain a mean value of 1 before the point of no return. To conclude, EnKF and PF give a weak but correct signal regarding eutrophication throughout their simulation time, while MCMC begins with a false signal but quickly predicts a high probability as the transition draws near.

Similar patterns of performance are observed when the methods are tested for two alternative strategies (see S4 Text). Two alternative strategies were tested: a linear increase in anthropogenic phosphorus inputs across the entire time period, and an abrupt increase in anthropogenic phosphorus inputs from low to high at year 40. All data assimilation methods show similar performance in the alternative strategies as the step-wise high emissions strategy. EnKF and PF both predict an increasing probability eutrophication, similar to the step-wise increase case discussed before. MCMC’s behavior is also similar, with an initial decrease in estimated probability of eutrophication followed by an increase.

We find that although there is considerable uncertainty in estimation of model parameters, *P*_*crit*_ is relatively well estimated by EnKF, PF, and MCMC if all learning were to stop at the point of no return (Fig 5). This is shown in Fig 6, which shows the probability density functions (PDFs) estimated by each method for the phosphorus concentrations at the last time step, model parameters, and *P*_*crit*_ at the point of no return. Since it is possible to attain similar *P*_*crit*_ values across different combinations of parameters, high uncertainty in parameters does not necessarily translate to high uncertainty in the more decision-relevant parameter, *P*_*crit*_ [32]. There are considerable uncertainties in parameter estimates for all methods, as shown by the spread of the PDFs. Overall, PF and MCMC show a close match between the assumed true values of loss and recycle parameters while higher biases are observed for EnKF. In fact, the mode of the MCMC-estimated PDF for *P*_*crit*_ is the closest to the true values. PF (EnKF) estimates the mode of *P*_*crit*_ at slightly lower (higher) values. Another interesting feature is that EnKF, PF, and MCMC are able to predict the high concentrations of phosphorus in the lake at the end of the time period, while the mode of PC is at the lower end with a considerably large spread.

### Discussion

The time evolutions of learning trajectories of each method showed a sharp convergence to true parameter estimates as the system approaches eutrophication (Fig 2). Thus, while eutrophication itself is undesirable, the observations made as the system nears the eutrophic state have a much higher information content than those that are made far from it. If adequate data assimilation methods are employed, it may be possible to gain sufficient information about the critical threshold to prevent eutrophication. We test this by projecting the system states based on knowledge about model parameters gained by using observations up to the point of no return. We found that it is in fact possible to reduce uncertainties regarding model parameters sufficiently to confidently predict eutrophication under presently employed anthropogenic phosphorus emission strategies (Fig 5). Thus, a decision maker will have the knowledge that if the phosphorus emissions trajectory is maintained, eutrophication is most probable. This should make the decision maker to change the phosphorus emissions strategies.

We also found that in the case of the low-emissions strategy, neither of the data assimilation schemes were able to identify the true model parameters. This was attributed to the low information content in the observations of the low-emissions case (Fig 3). However, this did not prevent the methods from converging to the true probability of eutrophication, i.e., 0 for the low emissions case (Fig R in S1 File). Even though the lake’s dynamics are poorly characterized, the emissions are low enough to prevent eutrophication across a range of model parameters. Thus, knowledge of the true parameter values is not necessary to predict the true state as the emissions strategy dominates the lake’s behavior. Note that, however, some methods (PF and EnKF) do predict a large range of probability of eutrophication up to year 40, even for the low emissions case.

Overall, we find a tradeoff between the ability to learn about the system and the ability to prevent an irreversible transition to an undesirable state. It might be worth pointing out that controlled increases in emissions may actually reveal more information about the system and may subsequently lead to better management strategies. This is shown to be conceptually feasible for the stylized model used in our analysis. However, the lake model is a rather simple model with low computational requirements. Even for this simple and computationally inexpensive model, our results show that the number of ensembles used by an assimilation scheme significantly affects its performance (Fig 4). To what extent these data assimilation methods can improve learning about real systems that are characterized by irreversible dynamics is worth exploring in future research.

Another interesting result is the convergence of all Bayesian approaches to the true values of *P*_*crit*_ despite lack of convergence to true parameter values (Fig 6). The observed convergence of methods is a consequence of the structure of the lake model. Different values of the loss and recycling parameters combine to lead to similar values of *P*_*crit*_ [32]. Thus, a method may converge to different regions of model parameters that predict similar eutrophication dynamics. This phenomenon, where multiple possible physical conditions of the system may lead to the same observed response behavior, is termed ‘equifinality’ [46]. Equifinality of model parameters affects the manner in which data assimilation methods interpret the information embedded in the observations. The observations allow the methods to identify the true *P*_*crit*_ but do not necessarily have sufficient information to identify true values of the model parameters. Incidentally, it is the true value of *P*_*crit*_ that guides decision making for the lake problem and therefore, this equifinality of model parameters does not necessarily lead to poor decisions.

There is a large body of research on advancing data assimilation techniques and several versions of each filter exist in the literature [47]. Our analysis (and the associated computer code) implements the most commonly described version of each filter (as well as MCMC) and provides a useful test-bed. We present an overview and test of different data-model fusion approaches that could be used to guide adaptive decision making in the face of potential threshold responses. For simplicity, we made several assumptions and simplifications that point to future research needs. A key simplification is the assumption that the process noise is zero. In reality, it is possible that the real system needs to be studied as a stochastic process and this process noise needs to be accounted for. Although not attempted here, PF and MCMC can include system noise, provided likelihood functions are adequately defined in MCMC and PF. Although the process noise in our model setup is assumed to be zero, the measurement noise included in the analysis may also be interpreted as the combined effects of observation error and unresolved internal variability of the system [4, 44, 45]. Other studies employ a similar setup using MCMC methods to better understand learning dynamics regarding climate thresholds [4, 44]. Furthermore, our analysis considers only a small subset of the relevant uncertainties and strategies. Additional uncertainties may arise due to the random nature of anthropogenic inputs to the lake, and the structure of the lake model. It will be interesting to explore the joint consequences of other parameter choices, model structures, and strategies in future work.

## Conclusions

Our results can help to better understand the conclusions from previous analyses by [9] with respect to learning about a tipping point. In contrast to our study design that tracks uncertainties regarding model parameters, [9] tracks the belief in two different formulations of the lake, one of which results in eutrophication while the other does not. The true model is a combination of both models and a critical threshold is used to decide which of the two is true at a given time. In their setup, the decision maker is never allowed to learn about the true model and reverts back to believing with certainty that one model was correct even though prior experience indicates it was not. This work provides an important analysis of the impact of structural model uncertainty on optimal management strategies using a simple and transparent data-model fusion technique and demonstrates that learning can be crucial. Building on these insights, [10] explored learning about parametric model uncertainty using the Kalman filter to update the knowledge regarding the lake model’s parameters.

Our study shows that increasing the complexity (and also power) of data-model fusion techniques can improve the ability to learn about the parameters (and by extension the model structure) in some cases, but the warning signs can be faint and late (Fig 5). Thus, decision makers in this simple test case might face a relatively small window between learning and the point of no return. In addition, we demonstrate and quantify how the choice of management strategy affects the learning rate. We find that some policy schemes lead to a monotonic change in predicted probability of eutrophication over time, while others do not. For example, managers employing EnKF and PF may use the signal of increasing probability of eutrophication to adopt a pollution strategy with low pollution levels. On the other hand, decision makers using MCMC techniques may respond the initial signal of a low eutrophication probability by increasing pollution, but as the transition approaches, they are likely to learn relatively quickly about the lake’s true parameters and choose to adjust the policies accordingly.

Another interesting aspect of our results is the convergence to true *P*_*crit*_ values of the EnKF, PF, and MCMC methods before the point of no return (Fig 6). A recent study by [32] suggests that decision makers may be more interested in accurate estimates of *P*_*crit*_ as opposed to individual values of the lake model parameters. This is because it represents an integrated measure of the lake’s ability to avert eutrophication. The fact that three out of the four methods considered here converge to *P*_*crit*_ values before the point of no return indicates that there is a small but finite window of opportunity for lake managers to avert eutrophication.

## Supporting information

S1 File This supplemenatary material contains all supporting figures and their captions. (PDF)

S1 Text Description of data assimilation methods. Details on the Ensemble Kalman filter, Particle filter, and Markov Chain Monte Carlo method.
(PDF)

S2 Text Sample depletion. Discussoin on the sample depletion issue for the Ensemble Kalman filter, Particle filter, and Precalibration.
(PDF)

S3 Text Confidence interval estimation. Estimation of 90% confidence intervals from ensembles for all data assimilation methods.
(PDF)

S4 Text Testing alternative strategies. Testing two additional alternative high emission anthropogenic pollution strategies. Both alternative strategies result in eutrophication of the lake, but the timing and rate at which eutrophication occurs are different.
(PDF)
