# Supplementary material for: Skill (or lack thereof) of data-model fusion techniques to provide an early warning signal for an approaching tipping point
Source: PLoS One. 2018 Feb 1;13(2):e0191768. doi: 10.1371/journal.pone.0191768 (PMC5794081; doi:10.1371/journal.pone.0191768)
Supplement: S1 File — (PDF) [file pone.0191768.s001.pdf]

Supporting information

S1 File This supplemenatary material contains all supporting figures and their captions.

Fig. A Synthetic observations of phosphorus in the lake (gray lines) generated by adding noise to the assumed truth (blue line) for the high emissions case. True values and noisy observations are shown by solid blue lines and blue circles, respectively. Thick grey observations are used for the results in Figs. 2 6. All synthetic observations are used to assess the range of performance in Figs. 4 5 of the main text.

Fig. B Same as S1 but for the low emissions case. All synthetic observations are used to assess the range of performance in Fig. 4 of the main text.

Fig. C 90% Confidence intervals for phosphorus (black lines) using the EnKF for (a) low, and (b) high emission strategies. Confidence intervals are obtained using the formulae in S3 Text. After assimilating the observations at a time step, the forecast for the next time step constitutes the ensemble which is used to estimate CIs.

Fig. D Same as Fig. C, for PF.

Fig. E Same as Fig. C, for PC.

Fig. F Same as Fig. C, for MCMC.

Fig. G 90% Confidence intervals for loss parameter, b (black lines) using the EnKF for (a) low, and (b) high emission strategies.

Fig. H Same as Fig. G, for PF.

Fig. I Same as Fig. G, for PC.

Fig. J Same as Fig. G, for MCMC.

Fig. K 90% Confidence intervals for recylce parameter, q (black lines) using the EnKF for (a) low, and (b) high emission strategies.

Fig. L Same as Fig. K, for PF.

Fig. M Same as Fig. K, for PC.

Fig. N Same as Fig. K, for MCMC.

Fig. O Same as Figure 2b but for 5000 samples for MCMC as opposed to 991 in Figure 2b.

Fig. P The Kalman gain matrix for phosphorus in the lake, *b* parameter, and *q* parameter for EnKF output in Fig. 2b.

Fig. Q Estimates of probability density functions (PDFs) of phosphorus in the lake at t=100 years, loss parameter (b), and recycle parameter (q), as a function of learning time for the Metropolis-Hastings algorithm implementing MCMC. The density distribution is shown using the kernel density of the last 50% of the MCMC chain. Learning time in years is shown on the left hand side of each horizontal panel. The bandwidth for kernel density estimates is set equal to the standard deviation of the variable. Unscaled (scaled) bandwidths are shown by black (gray) curves. The bandwidth is scaled to aid visualization. Assumed true values of parameters and consequent values of phosphorus are shown as solid blue lines. Synthetic observations of phosphorus used to train MCMC are shown as blue circles.

Fig. R Same as Fig. 5 but for the low emissions strategy in the main text as shown in Fig. 1.

Fig. S The number of ensemble members (ensembles/particles/samples) remaining in the analysis for EnKF, PF, and PC, as a function of learning time. At each time step, samples are removed if they violate either of the following constraints: 1) the simulated states or parameters are negative, 2) loss parameter, b is not between 0 and 1, and 3) recycle parameter, q is not between 2 and 8.

Fig. T Two alternative strategies used to test the performance of learning methods.

Fig. U Same as Fig. 2 but for the linear increase in emissions strategy in Fig. T.

Fig. V Same as Fig. 2 but for the step increase in emissions strategy in Fig. T, where the step increase occurs at year 40.

Fig. W Same as Fig. 5 but for the linear increase emissions strategy in Fig. T.

Fig. X Same as Fig. 5 but for the step increase emissions strategy in Fig. T, where the step increase occurs at year 40.
