# Supplementary material for: Skill (or lack thereof) of data-model fusion techniques to provide an early warning signal for an approaching tipping point
Source: PLoS One. 2018 Feb 1;13(2):e0191768. doi: 10.1371/journal.pone.0191768 (PMC5794081; doi:10.1371/journal.pone.0191768)
Supplement: S1 Text — Details on the Ensemble Kalman filter, Particle filter, and Markov Chain Monte Carlo method. (PDF) [file pone.0191768.s002.pdf]

Supporting information

S1 Text Description of Data Assimilation Methods

S1.1 Ensemble Kalman filter (EnKF) A detailed overview of filtering methods is given in [1]. Here, we describe the implementation of the EnKF. The model forecast, denoted by superscript f, is combined with the observations to provide updated estimations of the state variable (analysis), denoted by superscript ana. The EnKF generalizes the Kalman filter that uses observations to update forecast from a linear model with normally distributed observation errors. Following [2],

$$x^{ana} = x_f + K(y - hx_f), \tag{1}$$

where x is the state variable, K is the Kalman gain, y is the observation vector, and h is a linear measurement function. The Kalman gain, is given by,

$$K = P^f h_t (hP^f h_T + W)^{-1}, \tag{2}$$

where P^f is the state error covariance matrix and W is the measurement error covariance matrix. Recall that in the case of the lake problem, W simplifies to ν, as only one variable is being observed. Using equation 11, the updated values of the state variable can be obtained. In a similar manner, the error covariance of the forecasted state, P^f is also updated to P^{ana} using:

$$P^{ana} = (I - KH)P^f. \tag{3}$$

The analyzed state is the optimal estimator, i.e., it minimizes least squared error of the estimates, if the model function $f(\cdot)$ is linear, the errors are independent and Gaussian. The model forecast for the next time steps is then obtained using,

$$x^f_{t+1} = fx^{ana}_t. \tag{4}$$

The EnKF generalizes the Kalman filter to a non-linear model by approximating the error covariance matrix using random realizations of model states, termed ensemble members. First, ensemble members representing a variety of model states are generated from an assumed prior distributions of the state variables. These ensemble members are propagated in time individually using the model function and updated using equation S1. Since the true state is unknown, the forecasted covariance matrix at any time step is approximated as,

$$P^f \simeq P^f_e = \overline{(x^f_e - \overline{x^f_e})(x^f_e - \overline{x^f_e})^T}, \forall e \in (1, ..., N_e), \tag{5}$$

where P^f_e is the covariance matrix estimated from the ensemble forecast, x^f_e is the forecasted states by the e_{th} ensemble member, $\bar{x}^f_e$ is the mean forecast across all ensemble members, and N_e is the number of ensemble members. The Kalman gain matrix is estimated using equation 12 by replacing the true forecast covariance matrix with its ensemble approximation. In a similar way, the true covariance of the analyzed state in equation 13 is replaced by its ensemble approximation:

$$P^{ana}_e = \overline{(x^{ana}_e - \overline{x^{ana}_e})(x^{ana}_e - \overline{x^{ana}_e})^T}. \tag{6}$$

Readers are directed to [2, 3] for more details on the ensemble Kalman filter.

S1.2 Particle filter (PF) The PF relaxes the assumption in the EnKF further by allowing the associated noise to be non-Gaussian in addition to being applicable for a nonlinear model. PF bases itself directly on the Bayesian recursion in equations (3)-(5) of the main text and approximates the posterior density $p(J_t|y_t)$ by a set of random samples,$(x_p^t, w_p^t)_{p=1}^{N_p}$, where x_p^t are the particles with associated weights w_p^t at time t, and the subscript represents the pth of N_p total particles. As with the EnKF, particles are initialized from an assumed prior distribution. Then, observations are used to estimate the likelihood $p(y_t|x_p^t, w_p^t)$, which when normalized by the sum of likelihoods across all particles yields the updated particle weight. The posterior density at time t is then approximated as,

$$P(J_t|y_t) \approx \sum_{p=1}^{N_p} w_p^t \delta(x_t - x_p^t), \tag{7}$$

where δ is the Dirac delta function and the remaining terms are defined before. By drawing with replacement from x_p^t with probabilities in w_p^t, the updated state is obtained. See [4–6] for further details. Note that as we implement a joint parameter-state estimation, in each iteration of EnKF and PF, the parameters are updated first followed by the state update.

S1.3 Markov Chain Monte Carlo (MCMC) Metropolis-Hastings Algorithm: The Metropolis-Hastings algorithm starts with a random sample in the joint state-parameter space [7]. Then, using a proposal distribution, it selects another sample in the space. The proposed sample is accepted or rejected based on the ratio of the posterior density of the proposed sample to that of the original sample if the proposal distribution is symmetric (Metropolis algorithm). If the proposal distribution is asymmetric, the ratio of posterior densities is multiplied by the ratio of conditional probability of proposing the new state given the old state and vice-versa (Metropolis-Hastings algorithm). The calculation of posterior density requires estimation of likelihood where observations are assimilated into the algorithm. The design of the algorithm allows it to spend more time in high posterior density regions as it samples the space. Consequently, the sequence of samples (MCMC chain) better approximates the posterior as chain size increases. In order to eliminate the impact of starting location on the approximation, we retain only the second half of the chain. See [8] for further details on the algorithm.

Here, we apply the Metropolis algorithm available in the R-package mcmc using the metrop function [9,10]. The function takes as inputs the initial location of the algorithm in the joint state-parameter space (Table 1), a function to estimate the posterior density of a sample, length of the chain, and a scale parameter that controls the step size of the proposal. In its default setting, the proposal is set to a multi-dimensional standard normal distribution (i.e., standard deviation =1), with the off-diagonal covariances set to 0. As each state/parameter is likely to have different levels of uncertainty associated with it, we set the scale to the guess estimate of the covariance matrix (Table 1), thus allowing lower step sizes for the state (phosphorus in the lake) and loss parameter but slightly higher step size for the recycle parameter.

In order to facilitate the comparison with EnKF and PF, we implement MCMC such that at each time step, only the observations until that time step are used to estimate the posterior. Thus, the forecasts for time step, t, require running the algorithm t*(t-1)/2 times. As with other methods, MCMC also needs specification of the number of random walks through the joint state-parameter space (chain size), and is likely to converge only after a sufficient number of steps are taken. As this is difficult to ascertain prior to the run, we estimate the impact of this choice on performance in the results. As a result, a 100 year run with a chain size of 10 results in

10*(100)*(100)/2=50,500 function evaluations. Each function evaluation is one run of the lake model. Thus, the x-axis in Fig. 4 is in multiples of 50.50 for MCMC. Fig. Q in S1 File shows the time evolution of MCMC's estimates of lake's phosphorus states at t=100 and model parameters.

References

1. Law K, Stuart A, Zygalakis K. Data assimilation: a mathematical introduction. vol. 62. Springer; 2015.

2. Burgers G, Jan van Leeuwen P, Evensen G. Analysis scheme in the ensemble Kalman filter. Monthly Weather Review. 1998;126(6):1719–1724.

3. Evensen G. The ensemble Kalman filter: Theoretical formulation and practical implementation. Ocean Dynamics. 2003;53(4):343–367.

4. Gordon NJ, Salmond DJ, Smith AF. Novel approach to nonlinear/non-Gaussian Bayesian state estimation. In: IEE Proceedings F (Radar and Signal Processing). vol. 140. IET; 1993. p. 107–113.

5. Kitagawa G. Monte Carlo filter and smoother for non-Gaussian nonlinear state space models. Journal of Computational and Graphical Statistics. 1996;5(1):1–25.

6. Bergman N. Recursive Bayesian estimation. Department of Electrical Engineering, Linköping University, Linköping Studies in Science and Technology Doctoral dissertation. 1999;579:11.

7. Metropolis N, Rosenbluth AW, Rosenbluth MN, Teller AH, Teller E. Equation of state calculations by fast computing machines. The Journal of Chemical Physics. 1953;21(6):1087–1092.

8. Chib S, Greenberg E. Understanding the Metropolis-Hastings algorithm. The American Statistician. 1995;49(4):327–335.

9. Geyer CJ, Johnson LT. mcmc: Markov Chain Monte Carlo. R package version 09-2, URL http://CRAN R-project org/package= mcmc. 2013;.

10. Team RC. R: A language and environment for statistical computing. R Foundation for Statistical Computing, Vienna, Austria. URL http://www. R-project. org. Accessed (June 25, 2015). 2015;.
