# Supplementary material for: Skill (or lack thereof) of data-model fusion techniques to provide an early warning signal for an approaching tipping point
Source: PLoS One. 2018 Feb 1;13(2):e0191768. doi: 10.1371/journal.pone.0191768 (PMC5794081; doi:10.1371/journal.pone.0191768)
Supplement: S2 Text — Discussoin on the sample depletion issue for the Ensemble Kalman filter, Particle filter, and Precalibration. (PDF) [file pone.0191768.s003.pdf]

Supporting information

S2 Text Sample Depletion As mentioned in the main text, we screen state-parameter sets at each iteration to remove those that are physically unrealistic for EnKF, PF and PC. The number of samples remaining at a time step after removing these combinations is plotted in Fig. S in S1 File. The figure shows that EnKF is most affected by this followed by PC and PF. By the end of 100 years, EnKF, PF, and PC are left with 54%, 85%, and 85% of their original samples for the low emissions case, respectively. For the high emissions case, these reduce to 1%, 85%, and 0.5% for EnKF, PF, and PC, respectively. This indicates severe sample deterioration for EnKF as compared to PF. Despite this, EnKF still emerges as the fastest converging method. Thus, our overall results are likely to favor EnKF even more if the issue of sample deterioration is accounted for in the algorithm such that no unrealistic parameter combinations are created during random generation. For MCMC, we implement this by making the likelihood function a large negative number in case of physical violations, thus those regions are not explored further by the algorithm.
