# Supplementary material for: Skill (or lack thereof) of data-model fusion techniques to provide an early warning signal for an approaching tipping point
Source: PLoS One. 2018 Feb 1;13(2):e0191768. doi: 10.1371/journal.pone.0191768 (PMC5794081; doi:10.1371/journal.pone.0191768)
Supplement: S3 Text — Estimation of 90% confidence intervals from ensembles for all data assimilation methods. (PDF) [file pone.0191768.s004.pdf]

Supporting information

S3 Text Confidence Interval Estimation The formula used to estimate 90% confidence intervals from ensembles comprising EnKF, PF, PC, and MCMC output is,

$$CI_{90} = \hat{x}_t \pm \sqrt{var(x_k)} Z_{95}, \tag{1}$$

$$\hat{x}_t = \sum_{ens=1}^{N_t^{ens}} x_t^{ens}, \tag{2}$$

$$\hat{x}_t = \sum_{ens=1}^{N_t^{ens}} (x_t^{ens} - \hat{x}_t)(x_t^{ens} - \hat{x}_t)^T, \tag{3}$$

where $\hat{x}_t(\sigma_{x_k})$ is the mean (variance) of the ensemble, x_t^{ens} is each member of the ensemble of size N_t^{ens} at time t. Z_{95} is the standard normal variate evaluated at 0.95 for 90% CI (1.645). Note that for PF, the formula is applied after all the weights are equalized, so weights are not considered in the equations above. Figs. C-N in S1 File show the estimated CIs for each data assimilation method.
