# Supplementary material for: Skill (or lack thereof) of data-model fusion techniques to provide an early warning signal for an approaching tipping point
Source: PLoS One. 2018 Feb 1;13(2):e0191768. doi: 10.1371/journal.pone.0191768 (PMC5794081; doi:10.1371/journal.pone.0191768)
Supplement: S4 Text — Testing two additional alternative high emission anthropogenic pollution strategies. Both alternative strategies result in eutrophication of the lake, but the timing and rate at which eutrophication occurs are different. (PDF) [file pone.0191768.s005.pdf]

Supporting information

S4 Text Testing Alternative Strategies This section reproduces the results from the main text for two alternative high emission anthropogenic pollution strategies. Both alternative strategies result in eutrophication of the lake, but the timing and rate at which eutrophication occurs are different. These are (Fig. T in S1 File):

1. An emissions strategy that increases from 0.00 to 0.20 linearly throughout the time period as opposed to increasing in steps of 5 years as in the high emissions strategy in the main text.

2. A step increase in emissions from 0.05 to 0.20 at year 40. Emissions are fixed at 0.05, and 0.20 before and after year 40, respectively.

   The mean trajectories for each method are shown in Figs. U-V in S1 File. For each strategy, the parameter trajectories eventually converge to true parameter estimates, similar to the pattern in the Fig. 2 of the main text. However, the timing of convergence depends on the type of strategy employed. The probability of eutrophication as predicted by each method as a function of time for the strategies is plotted in Figs. W-X in S1 File, which are designed similar to Fig. 5 of the main text. Both strategies show similar patterns of predicted probability of eutrophication as the step-wise high emissions strategy of the main text. EnKF, followed by PF are able to track the assumed truth closely throughout the learning period. MCMC's degradation in the initial periods is the same across all strategies, though after the first few decades, MCMC is also able to predict more than 50% probability of eutrophication.
